# Low dose thirdhand smoke exposure enhances platelet functional responses in mice

**DOI:** 10.3389/ebm.2026.10679

**Published:** 2026-03-12

**Authors:** Precious O. Badejo, Ahmed B. Alarabi, Hamdy E. A. Ali, Lanam Millican, Reina De La Paz, Shelby S. Umphres, Sadia Kamal, Fatima Z. Alshbool, Fadi T. Khasawneh

**Affiliations:** 1 Department of Pharmaceutical Sciences, Irma Lerma Rangel College of Pharmacy, Texas A&M University, Kingsville, TX, United States; 2 Irma Lerma Rangel College of Pharmacy, Texas A&M University, Kingsville, TX, United States; 3 Department of Pharmacy Practice, Irma Lerma Rangel College of Pharmacy, Texas A&M University, Kingsville, TX, United States

**Keywords:** cardiovascular disease, novel tobacco exposure, platelets, thirdhand smoke, thrombosis

## Abstract

Although cigarette smoking is the most preventable cause of cardiovascular diseases, most researchers have focused on either direct/firsthand or secondhand smoke exposures. Recently though, attention has shifted to an emerging/indirect exposure trend-known as thirdhand smoke (THS)- which was previously “overlooked.” This phenomenon, which was/is thought to be harmless, has been identified as a serious health risk, including in the context of thrombogenesis/platelets. However, whether low dose THS exposure has the capacity to modulate platelets has not been investigated. Two sets of household materials were exposed to 20 cigarettes/day for a week on an alternating basis, with controls exposed to clean air. After the first set of exposed materials is placed in mice cages, exposure of the second set is initiated. The materials were interchanged weekly, for a total exposure duration of 1 month. Mice were then subjected to multiple platelet function assays. THS exposed mice exhibited shortened tail bleeding and occlusion times, indicating a prothrombotic phenotype. Moreover, we also observed that platelets from the exposed mice exhibited an enhanced aggregation response. However, we did not observe any gender differences in our *in vivo* as well as aggregation experiments; hence, subsequent characterization was carried out on male mice. It was also found that dense granules release, integrin activation, and PS exposure were also potentiated in the exposed platelets compared to the controls. Finally, we observed for the first time that the tobacco-specific nitrosamine and THS toxicant NNK enhanced platelet aggregation and thrombus formation. Collectively, we provide documentation that low dose of THS exposure is detrimental to health by increasing the risk of thrombosis through a hyperactive platelet phenotype that involves the toxicant NNK.

## Impact statement

We document for the very first time that exposure to THS, even under low dose conditions, could modulate platelets hemostatic responses, thereby promoting a thrombogenic phenotype in exposed mice. This suggests that even a relatively short and small amount of exposure can be extremely harmful, which is alarming for susceptible populations who are mostly unaware when exposed. Importantly, THS phenomenon-albeit indirect-is viewed as harmless with little/no attention paid to it. Our work also highlights the impact that this preventable risk factor has on the leading cause of death worldwide, namely cardiovascular disease. Bearing in mind that mice tobacco studies have been shown to “map” very well with humans, we believe that our studies should serve as a guide for future research, public enlightenment and policy guidance. We aim to educate the public on these health risks which could lead to reduced disease states and mortality caused from smoking.

## Introduction

Cigarette smoking, which remains the single most preventable cause of death globally, accounts for approximately 10 years reduction in the life expectancy of smokers when compared to non-smokers [1]. In fact, in 2019, a report by the World Health Organization (WHO) revealed that tobacco products kill about 50% of users, which accounted for more than 8 million deaths annually [2]. Among these, 7 million died because of direct exposure or firsthand smoke (FHS), while 1.2 million deaths were attributed to secondhand smoke (SHS) [2]. Additionally, a more recent report from the National Health Interview Survey (NHIS) in 2021 revealed that 46 million US adults were current tobacco users with the highest percentage using traditional cigarettes [3]. Smoking is associated with a range of cardiovascular diseases (CVDs), including heart failure, and atherosclerosis [4–6]. Additionally, multiple studies documented that cigarette smoking could modulate platelet reactivity, resulting in thrombotic episodes [7]. To this end, the toxicant profile of the smoke of cigarettes-which is responsible for the cigarette smoke-associated disease phenotypes-involves over 7,000 toxicants, including nicotine, carbon monoxide and tar. These, amongst potentially other toxicants, contribute to the pathogenesis of CVDs by inducing inflammation, endothelial dysfunction, and thrombus formation [8, 9]. As such, there is growing interest in investigating the contribution of other tobacco/THS toxicants to the thrombosis phenotype, such as tobacco-specific nitrosamines, including NNK [(4-(methylnitrosamino)-1-(3-pyridyl)-1-butanone]. Indeed, our findings indicate that NNK could enhance platelet reactivity and thus might contribute to the prothrombotic impact of third-hand smoke exposures.

In this connection, “third hand smoke” (THS), which is an emerging health risk, has been a topic of interest and continues to be studied by several research groups, not only in the USA, but also worldwide. THS is defined as tobacco smoke residues and contaminants that may linger on smokers’ clothing, skin, and hair as well as carpets, curtains long after exposure to SHS [10, 11]. THS is difficult to eliminate and can remain embedded in indoor environments—such as homes and vehicles—for months after smoking has ceased [12, 13]. To this end, a number of reports confirmed significant levels of THS are present in the cars and residence of non-smokers whose houses were previously occupied by smokers [12–14]. Consequently, THS has been established as playing a significant part in exposure of non-smokers to tobacco products, even many years after smoking cessation in homes of smokers [15]. This is attributed, at least in part, to the fact that exposure to THS creates secondary toxins that remain “indoors”, even when doors and windows are opened to eliminate the “stench” [11, 16]. Additionally, the concentration of these toxins continues to increase in the blood, gradually over time, which further increases the dangers of THS to health due to this continuous exposure [17]. This theory was first described at a Children’s hospital located in Boston in 2009 [18]. Indeed, recent data points to the fact that THS can impair development, cause cytotoxicity, as well as DNA damage, which increases the risk of cancer, in mice [19].

With regards to sex differences in cigarette use, men generally use tobacco products more than women, with about 942 million men reportedly smoking globally relative to 175 million women. Although smoking has been dominated by men for decades, the rate of cigarette usage has escalated among women. According to a WHO report on prevalence/future tobacco use from 2000–2025, the rate of use was highest among European women (19%) globally. Even with the decline in smoking worldwide, it is projected that in 2025, the prevalence in women who smoke would be 18%, which is quite worrisome. An interesting body of evidence revealed that smoking affects men and women differentially in the context of CVD and lung diseases, with the risk of lung cancer found to be elevated in women more than in men [20]. Regarding CVDs, cigarette smoking significantly increased risk of coronary artery disease by 25% in women as compared to men [21]. However, these reports were based on direct cigarette exposure. To this end, we have previously documented that *in utero*, as well as long-, and short-term exposure to THS increases the risk of thrombosis [22–24]. Regarding *in utero* THS, our previous study revealed sex-related differences in integrin activation, dense and α-granules secretions, as well as PS exposure, whereas no difference were observed in bleeding and occlusion times. Moreover, sex differences were also observed in our THS 3-month study in which we used 40 cigarettes/day exposure protocol [24]. Additionally, our very recent study showed that even when the exposure to 40 cigarettes/day is for 1 month, THS can still exert occlusive effects [25]. In that study, our findings show a shortened bleeding and occlusion times in the exposed male and female mice, which was more striking in the females. However, whether these effects manifest under low “cigarette dose” experimental settings are yet to be determined.

Based on these considerations, we sought to investigate the effects of low dose THS, namely 20 cigarettes/day, on platelet reactivity and thrombus formation, in a sex-dependent manner under short term/1 month exposure settings [24].

## Materials and methods

### Thirdhand smoke (THS) exposure protocol

**FIGURE 1:**
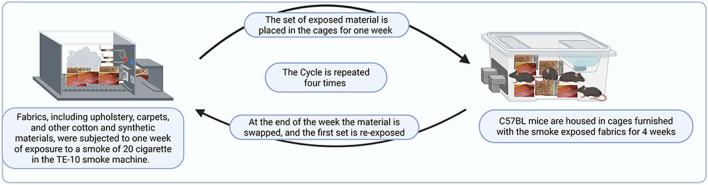
Illustration of the THS exposure protocol.

Mice were randomly divided into 2 groups and exposed to either THS or clean air as previously described [22]. Materials, which included upholstery (12in × 3.5in), cotton (7.5in × 5.5in), and square-sized carpets (2in × 2in), were exposed to cigarette smoke by being placed in the smoking machine. The exposure duration was for 1 month (4 weeks), using low dose/20 cigarettes/day for 1 week, and involved two sets of material, such that the exposure of the second set is initiated at the same time the first one is placed into the cages (after 1 week) to start the exposure. At the end of the 1 week of exposure, the material is swapped, and the first set is re-exposed, and this cycle is repeated (please see Figure 1 for an illustration of the exposure protocol). In other words, employing two sets of material that were exposed on an alternating-week basis ensures that mice are constantly exposed to THS or clean air. Of note, mice were exposed starting at 6 weeks of age, and the exposure was conducted on each sex separately.

### Reagents and materials

Thrombin and stir bars were purchased from Chronolog Corporation (Havertown, PA), whereas adenosine diphosphate (ADP), Avertine [(2,2,2-Tribromoethanol) and ferric chloride] were from Sigma Aldrich (St Louis, MO). The antibody for P-Selectin (Fluorescein isothiocyanate/FITC-conjugated P-selectin) was purchased from Cell Signaling Technology, Inc. (Danvers, MA), phycoerythrin-conjugated GPIIb-IIIa (αIIbβ3) antibody was from Emfret Analytics (Würzburg, Germany) and Annexin V (Phosphatidylserine PS) antibody was purchased from BD BioSciences (Franklin Lakes, NJ). The cotinine enzyme-linked immunosorbent assay (ELISA) kit was purchased from Calbiotech (El Cajon, CA). NNK was purchased from MedChemExpress (Monmouth Junction, NJ).

### Animals

C57BL/6J (6-week-old male) mice were purchased from the Jackson Laboratory (Bar Harbor, ME) and housed 5 mice per cage under 12/12 light/dark cycles. Each cage had uninterrupted access to water and food except when mice were being exposed. All protocols were approved by the Institutional Animal Care and Use Committee of Texas A&M University, College Station.

### Cotinine assay

To ensure that our THS exposure protocol systemically and effectively exposes mice to THS toxicants, we measured the serum levels of cotinine, a metabolite of nicotine, in both the THS and clean air–exposed mice, using the Cotinine Direct ELISA kit as per the manufacturer’s instructions.

### Tail bleeding time

The tail bleeding time assay was carried out as we described in another report [26]. Briefly, mice exposed to either THS or clean air for 1 month were anesthetized using 2.5% isoflurane. The tail of each mouse was cut 5 mm from the tip using a sterile scalpel and placed in 37 °C saline solution and the bleeding time was recorded until the bleeding stopped.

### *In vivo* FeCl_3_ carotid artery injury–induced thrombosis model

The *in vivo* thrombosis model was performed as described previously [26]. Thus, both THS and clean air–exposed mice were anesthetized with avertin, and the left carotid artery was isolated. Thereafter, 1 µL of 7.5% ferric chloride was applied to a 1 mm in diameter filter paper disc, which was immediately placed on top of the artery for 3 min. The Transonic Micro flow probe was used to establish the occlusion time.

### Mouse platelet rich plasma (PRP) preparation

Blood was collected from the heart of each mouse after anesthesia using 0.38% w/v sodium citrate solution as the anti-coagulant (Fisher Scientific, Hampton, NH). The pooled blood sample for each group was centrifuged at 180 g for 11 min, and the supernatant was harvested (platelet-rich plasma; PRP). Platelets were counted with the HEMAVET 950FS Multi-species Hematology System before each experiment.

### Human blood and platelet rich plasma (PRP) preparation

Blood samples were obtained from healthy adult volunteers/donors (3 males, age range between 24 and 48 years) after approval by the Institutional Review Board at Texas A&M University (IRB Reference Number: IRB2020-0385D). Informed consent was provided by all participants prior to phlebotomy. Venous blood (3–10 mL) was collected into tubes containing either citrate-phosphate-dextrose (1:9 v/v) or Benzylsulfonyl-D-Arg-Pro-4-amidinobenzylamide (BAPA) as the anticoagulant. For PRP preparation, blood from each donor was centrifuged at 180 g for 11 min, and the supernatant was harvested (PRP). Platelets were counted with the HEMAVET 950FS Multi-species Hematology System and counts adjusted before each experiment.

### Mouse washed platelet preparation

Blood was collected from each mouse as described above. Equal amounts of blood were mixed with modified HEPES Tyrodes buffer of pH 7.4 in a 2 mL Eppendorf tube. This mixture was centrifuged at 180 × g for 7 min at room temperature. PRP was recovered and centrifuged a second time at 400 × g for 10 min at room temperature. The recovered pellets were resuspended in 1 mL of HEPES Tyrodes and centrifuged again at 400 g for 10 min. The washed pellets were resuspended in 1 mL of Tyrodes buffer, before platelets were counted using the HEMAVET 950FS Multi-species Hematology System and adjusted to the concentrations indicated elsewhere.

### *In vitro* platelet aggregation

The PRP from THS-, and clean air–exposed mice was activated with the agonists ADP (1 µM) or thrombin (0.05 U/mL). In a separate set of experiments using human PRP, platelets were activated with ADP (0.25 µM) or thrombin (0.025 U/mL). Aggregation was measured using a model 700 aggregometer with each experiment repeated at least 3 times with blood pooled from at least 5 mice each time.

### Dense granule release

PRP was placed in cuvettes at 37 °C, with the aggregometer set at 1,200 rpm, after which 12.5 µL of the luciferase mixture was added. Next, PRP was activated using the agonists ADP (1 µM) or thrombin (0.05 U/mL). Release of ATP was measured using a model 700 aggregometer. Each experiment was repeated at least 3 times with blood pooled from at least 5 mice each time.

### Flow cytometric analysis

Briefly, washed platelets from clean air or THS exposed male mice were prepared as described above and 1 µL of 1 mM Ca^2+^ was added to Eppendorf tubes in triplicates for both groups. Afterwards, washed platelets at a concentration of 100,000 platelets/μL was added and incubated with FITC-conjugated CD62P (P-selectin), Annexin V (PS exposure) or JON/A (integrin activation) antibodies at room temperature for 30 min in the dark. Finally, platelets were stimulated with the agonist thrombin (0.5 U/ml) and fluorescent intensities were measured using a BD Accuri C6 flow cytometer. Each experiment was repeated 3 times from pooled blood samples of 5 mice for each group.

### Platelet thrombus formation measurement

Platelet thrombus formation under flow conditions was evaluated using the T-TAS01 system (Fujimori Kogyo, Japan) as described before [27]. Briefly, 400 µL of whole blood from healthy donors, anticoagulated with 50 μM BAPA, was incubated for 5 min with vehicle Dimethyl sulfoxide (DMSO) or 2 µM NNK. The treated blood was then perfused through PL-chip collagen-coated microchannels at a shear rate of 1,500 s^−1. The increase in flow pressure across the microchip was recorded as a measure of platelet thrombus formation, and platelet thrombogenicity was quantified as the area under the flow pressure curve (AUC) over a 10 min period.

### Immunoblotting

Washed platelets were prepared before being stimulated with thrombin (0.1 U/mL) for 3 min and lysed in a suitable volume of sample buffer (1×, BioRad). About 15 μg of protein lysates prepared from the clean air- and the THS- exposed groups were resolved onto a 4–20% SDS-PAGE gel (Bio-Rad, Hercules, CA) under reducing conditions. The fractionated proteins were transferred onto nitrocellulose membranes (Bio-Rad, Hercules, CA) and subsequently blocked with 5% bovine serum albumin for 1 h at room temperature. The blocked membranes were incubated overnight at 4 °C with primary antibodies against total/phosphorylated Akt, total/phosphorylated ERK, and β-actin. Total Akt, ERK, and β-actin, were used as an internal protein loading control. The membranes were washed thoroughly and incubated with the proper HRP-conjugated secondary antibodies for 1 h at room temperature and detected using ECL (Thermo Scientific, Rockford, IL). Images were obtained with ChemiDoc MP Imaging System (Bio-Rad, Hercules, CA).

### Statistical analysis

All experimental analysis was carried out using GraphPad Prism Version 7. A normality test was performed before each analysis and based on this result, the Mann Whitney test was used to evaluate differences in bleeding and occlusion times; whereas the differences in aggregation and secretion were analyzed using Student’s t-test and represented as mean ± SEM. Flow cytometry data was analyzed using one-way ANOVA with Tukey’s multiple comparison test as *post hoc*. All sex differences analysis was analyzed using two-way ANOVA with Tukey’s multiple comparison test. Statistical significance was fixed at p < 0.05 for all analysis.

## Results

### THS exposure systemically delivers nicotine to exposed mice

**FIGURE 2:**
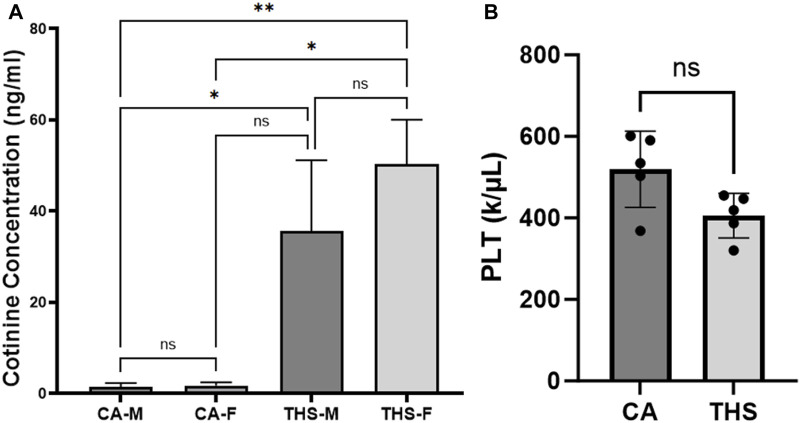
Low dose THS exposure results in systemic delivery of nicotine (cotinine) with no impact on platelet counts. **(A)** The concentrations of cotinine were measured in serum from THS and clean air–exposed mice. **(B)** Bar chart of platelet count showing comparison between THS vs. CA. Each bar represents the mean ± SD (**p < 0.01, *p < 0.05, NS: not significant).

Our data showed a significant increase in the level of cotinine in the THS exposed mice in comparison to clean air, in both males and females (Figure 2A). This confirms that our THS exposure model enables the delivery of toxicants, including nicotine to the mice’s circulatory system. Comparison of the levels of cotinine between the exposed male and female mice showed no statistical significance between the two sexes (Figure 2A), which is important when determining sex differences.

### THS exposure does not impact blood cell counts in mice

We first examined the total blood counts for the male and female mice and observed no difference in platelets and other blood cells from the THS-exposed and clean air groups (Table 1: data is presented as mean ± SEM; and Figure 2B). Blood cell count was assessed to rule out potential hematological effects that could confound interpretation of platelet functional outcomes. Specifically, alterations in platelet count, platelet size (MPV), or other blood cell populations could independently influence hemostasis and thrombosis, making it essential to confirm that the observed hyperactive platelet phenotype was not secondary to THS-induced changes in circulating cell numbers or bone marrow output [28]. Also, many platelet function assays are impacted by any change (drop) in platelet counts or quality [29]. Blood collected from the heart was analyzed using a Hemavet Hematology Analyzer. Representative data of platelet count comparison between THS vs. CA is shown in a bar chart in Figure 2B.

**TABLE 1:** Total blood counts obtained from THS and clean air exposed male and female mice.

Cell type	CA-M	THS-M	P-value	CA-F	THS-F	P-value
Platelets (k/µL)	488.5 ± 28.25	350.8 ± 90.31	0.20	230.3 ± 24.42	268.3 ± 13.38	0.20
MPV (fL)	3.88 ± 0.05	5.00 ± 0.66	0.14	4.29 ± 0.17	4.07 ± 0.05	0.24
RBC (M/µL)	9.61 ± 0.14	9.66 ± 0.21	0.87	8.9 ± 0.33	8.56 ± 0.31	0.50
LY (k/µL)	77.87 ± 1.44	76.88 ± 0.33	0.44	1.94 ± 0.18	2.11 ± 0.36	0.68
MO (k/µL)	4.56 ± 0.90	4.11 ± 0.31	0.65	3.08 ± 0.74	2.75 ± 0.30	0.69
WBC (k/µL)	4.81 ± 0.40	6.82 ± 1.33	0.20	2.30 ± 0.20	2.71 ± 0.47	0.44

### THS exposure modulates hemostasis in mice

**FIGURE 3:**
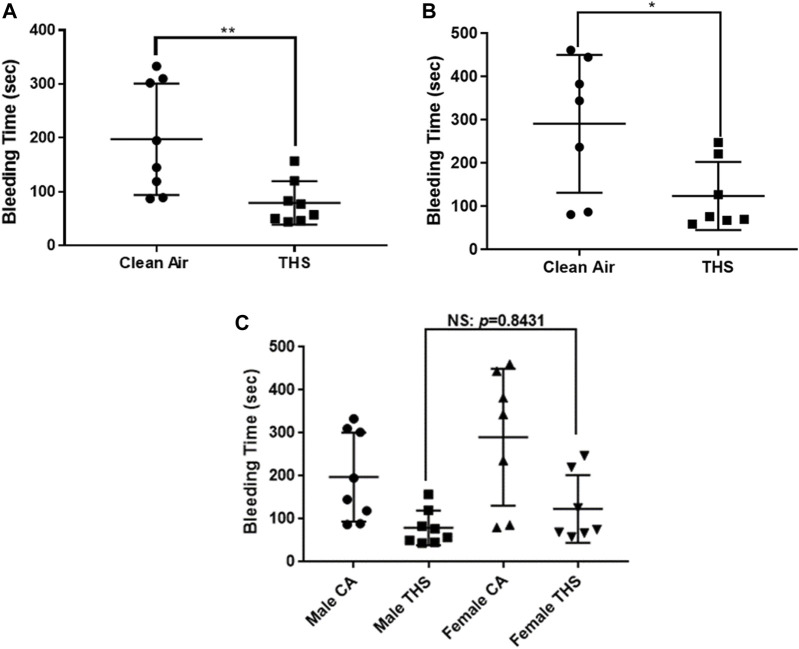
Low dose THS exposed mice exhibit shortened tail bleeding time, in both males and females. Both THS-exposed and clean air exposed male, n = 8 **(A)** and female, n = 7 **(B)** mice were anesthetized before being subjected to the tail bleeding time test. Each point represents the bleeding time from a single animal. Sex differences analysis is shown in panel **(C)**. Data was analyzed using Mann-Whitney test on GraphPad Prism version 7. (**p < 0.01, *p < 0.05, NS: not significant).

We next sought to determine the effect of our low dose exposure *in vivo* by investigating the hemostasis function of platelets utilizing the tail bleeding time test. As can be seen in Figures 3A,B, there is a significant decrease in the bleeding time in both male and female THS-exposed mice, respectively, in comparison with the clean air-exposed group, suggesting a platelet hyperactive phenotype. For the male group, the average bleeding time for THS-exposed was 79.25 ± 14.28 s, while the clean air exposed was 197.5 ± 14.28 s. For the females, THS-exposed mice had an average bleeding time of 123.1 ± 29.23 s relative to 290.1 ± 60.26 s in those exposed to clean-air. To assess sex differences, we compared the bleeding times between the male and female group. While the average bleeding time was slightly different (shorter in males relatives to females; Figure 3C), it did not reach statistical significance (*p* value of 0.8431).

### THS exposure modulates thrombosis in mice

**FIGURE 4:**
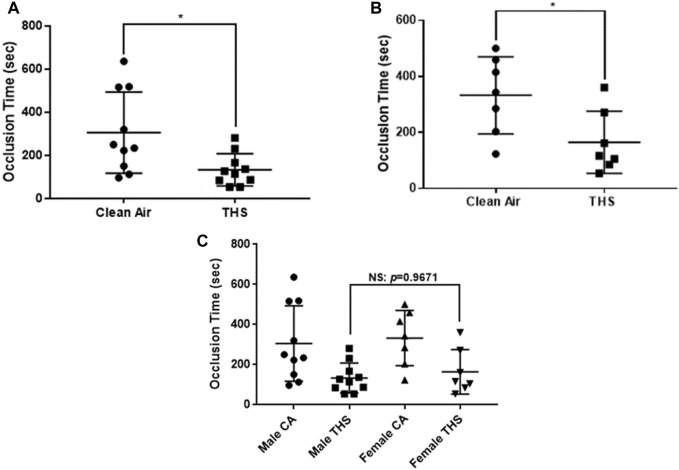
Low dose THS mice exhibit shortened occlusion time. Both THS-exposed and clean air exposed male, n = 10 **(A)** and female, n = 7 **(B)** mice were subjected to ferric chloride carotid artery thrombosis model. Sex differences analysis is shown in panel **(C)**. Data was analyzed using Mann-Whitney test on GraphPad Prism version 7. Each point represents the occlusion time from a single animal (*p < 0.05, NS: not significant).

Given that smoking is the main cause of cardiovascular mortality [8], we sought to investigate if our mice were prone to thrombosis by carrying out the ferric chloride carotid artery thrombosis model. Figures 4A,B show a reduction in the occlusion times of THS-exposed male and female mice as compared to clean air controls, respectively. For the male group, the average occlusion time for THS-exposed was 133.7 ± 23.56 s, while the clean air exposed was 305.8 ± 59.47 s. In terms of the females, THS-exposed mice had an average occlusion time of 164.6 ± 41.97 s, compared to an occlusion time of 332.6 ± 52.07 s in the clean-air exposed group. We also compared the occlusion times between the male and female group (Figure 4C) but did not observe any differences (*p* = 0.9671).

### THS exposure modulates in vitro platelet aggregation and dense granule secretion in mice

**FIGURE 5:**
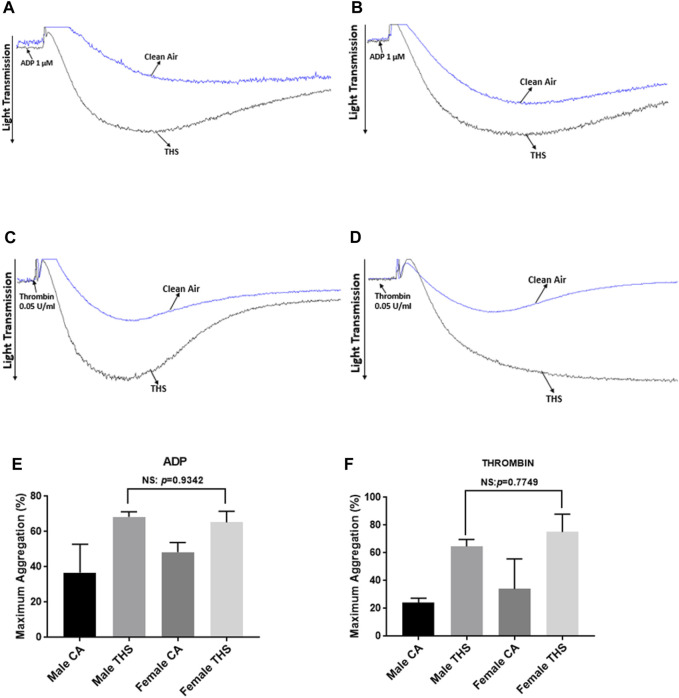
Low dose THS exposed mice show enhanced platelet aggregation. Both THS-exposed and clean air-exposed platelets from male **(A,C)** and female **(B,D)** mice were stimulated with either 1 µM ADP or 0.05 U/mL thrombin. Experiments were performed on at least 5 mice for each group and repeated at least twice. Sex differences as shown for ADP and thrombin in panel **(E, F)** respectively. Data was analyzed using student’s t-test on GraphPad Prism version 7.

**FIGURE 6:**
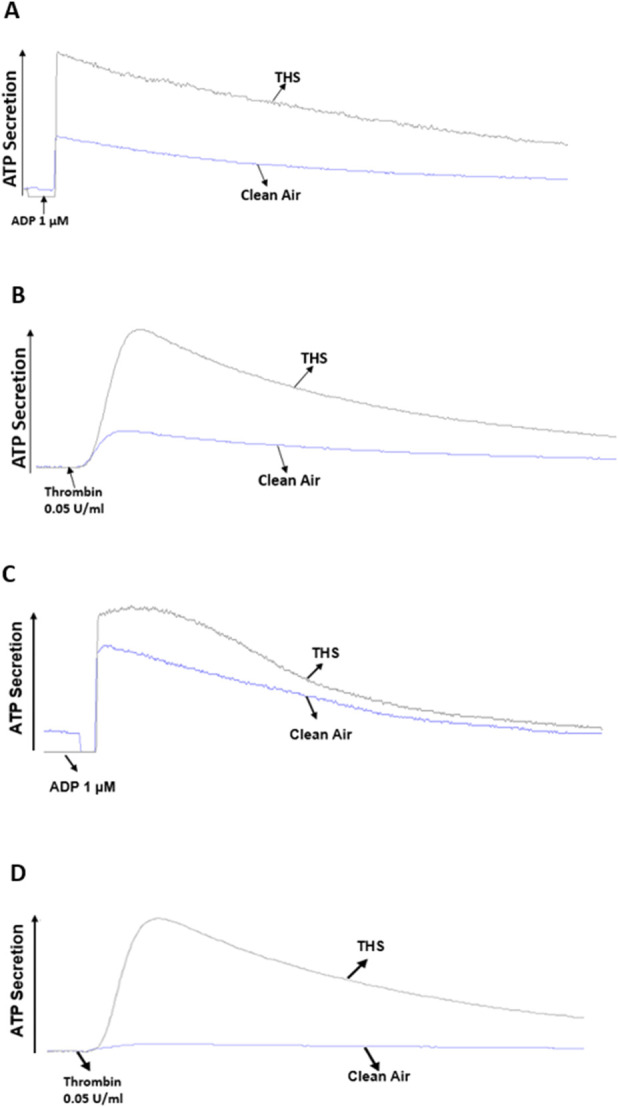
Low dose THS exposed mice show enhanced platelet dense granule secretion. Both THS and clean air exposed platelets from male **(A,B)** and female **(C, D)** mice were stimulated with either 1 µM ADP or 0.05 U/mL thrombin. Experiments were performed at least twice with pooled blood samples from at least 5 mice per group.

To determine if our THS low dose exposure would affect platelet function *in vitro*, we studied its effect on platelet functional responses, starting with platelet aggregation, which is the gold standard for assessing platelet function [30]. Our results show enhanced aggregation in the THS-exposed mice, in both males and females, in comparison to the clean air-exposed when platelets were stimulated with the agonists ADP (Figures 5A,B) and thrombin (Figures 5C,D); with data quantification for both sexes shown in Figures 5E,F, for ADP and thrombin, respectively. In line with the platelet aggregation result, dense granule release was also more enhanced in the THS-exposed platelets for male (Figures 6A,B) and female (Figures 6C,D) mice for both agonists. In terms of sex differences, we did not observe any, whether in the aggregation or dense granule secretion responses, as was the case with the *in vivo* phenotype.

### THS exposure increases integrin activation (αIIbβ3), α-granule secretion and phosphatidylserine expression in male mice

**FIGURE 7:**
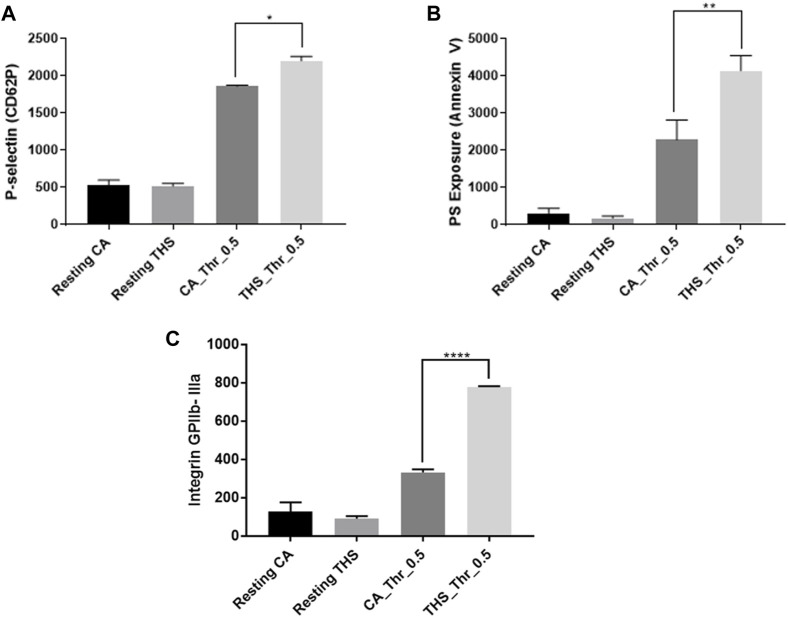
Low dose THS exposed mice show enhanced platelet activation markers. Both THS and clean air exposed platelets from male mice were stimulated with 0.5 U/mL of thrombin agonist before P-selectin **(A)**, PS exposure **(B)** and integrin GPIIb-IIIa **(C)** activation were assessed using flow cytometry. Experiments were performed at least twice with pooled blood from 5 male mice for each group (*p < 0.05, **p < 0.01, ****p < 0.0001). Data was analyzed using one-way ANOVA with Tukey’s multiple comparison test.

Based on our enhanced aggregation and dense granule results, we investigated if separate platelet activation markers were also enhanced due to the THS exposure. These experiments were performed only on male mice since we have not observed any gender differences *in vivo* or *in vitro* thus far. Using flow cytometry analysis, our results revealed that integrin activation, α-granule secretion and phosphatidylserine exposure were all enhanced in THS-exposed male mice in response to thrombin (Figures 7A–C). These results are all consistent with our *in vivo* results, suggesting a hyperactive platelet phenotype due to the exposure.

### NNK enhances platelet aggregation and *ex vivo* thrombus formation

**FIGURE 8:**
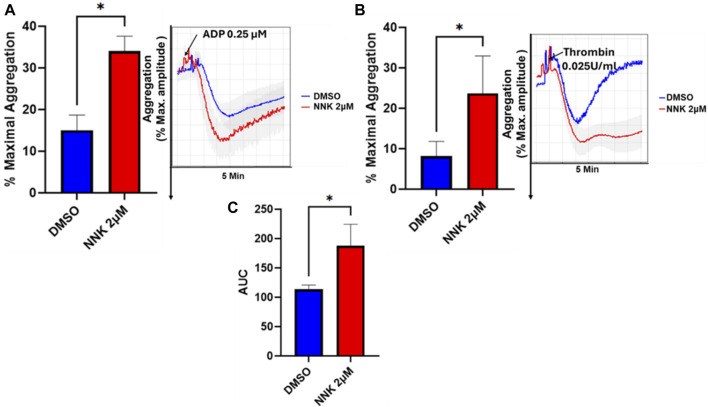
The THS toxicant NNK modulates platelet aggregation and platelets thrombus formation *ex vivo*. PRP or Blood was collected from healthy human subjects, treated with 2 µM NNK or the vehicle (DMSO) for 5 min before being subjected to either aggregation or T-TAS01 system analysis (PL chip flow under arterial shear stress conditions on a collagen coated surface). Platelet aggregation **(A,B)** was measured in response to stimulation with ADP **(A)** or thrombin **(B)**. The area under the curve/AUC10 comparing NNK vs. the vehicle DMSO was also assessed **(C)**. These data were obtained from healthy human subjects. Data were compared by running the student t-test using GraphPad Prism (*p < 0.05).

Although there has been interest in investigating the toxicants that underlie tobacco effects, including in the context of THS, with tar, carbon monoxide, and nicotine receiving significant attention, NNK has largely been ignored, especially concerning its role in CVD. Nonetheless, based on the published work [31] and our *in silico* analysis using the comparative toxicogenomic database (CTD) (not shown), NNK seems to be associated with pathways that might be involved in CVD. Our data showed, for the first time, that platelets incubated with NNK exhibit enhanced aggregation when activated by a low dose of ADP and thrombin (Figures 8A,B). Furthermore, NNK was also found to enhance thrombus formation *ex vivo*, as shown by the increase in the AUC using the T-TAS01 system (Figure 8C). Together, our results provide evidence that NNK is involved in instigating/contributes to a hyperactive platelet state as a result of exposure to THS.

### Low dose THS exposure enhance thrombin-induced Akt and ERK phosphorylation

**FIGURE 9:**
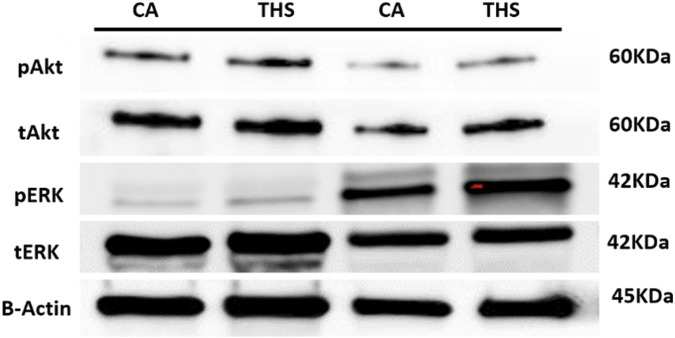
Low dose THS exposed mice show enhanced platelet Akt and ERK activation (phosphorylation). Platelets from low dose THS and clean air–exposed mice were prepared and washed. Platelets were either resting or stimulated with 0.1 U/mL thrombin for 3 min before being subjected to immunoblotting with anti-Akt, anti-pAkt, anti-ERK, and anti-pERK antibodies. Each experiment was repeated at least three times with blood pooled from a group of six to eight mice each time.

There is evidence that phosphorylation of Akt and ERK proteins is a critical signaling mechanism in platelet function and thrombus formation [32]. Therefore, we investigated whether THS exposure would enhance phosphorylation of Akt and ERK in platelets. Our data showed that Akt and ERK phosphorylation are indeed enhanced in the THS-exposed platelets, relative to controls after stimulation with thrombin (Figure 9). These results provide biochemical evidence and indicate even at low dose, Akt and ERK are an essential component in the THS-mediated modulation of platelets toward a hyperactive state.

## Discussion

The negative and public health consequences of indirect inhalation of cigarette smoke by non-smokers (and smokers) in the form of THS is yet to be fully understood and hence remains an active area of interest/investigation. Thus, while much progress has been made in the last several decades highlighting the pathogenesis associated with FHS and SHS, the same cannot be said regarding the adverse effects of THS [33]. One of the main differences between THS and other patterns of smoke exposure (FHS and SHS) is that while exposure to the latter is short and transient, THS exposure could go on for a very long period [34]. And the major pathways of exposure are thought to mainly be inhalation and uptake through skin and hair, when in contact with contaminated surfaces or clothing of smokers [17, 35]. The toxins emitted can remain on surfaces and react with other compounds to generate secondary pollutants, which could be released back into the air and inhaled [17]. Therefore, even the most vulnerable of our population-which includes toddlers and pregnant females who stay in houses with people who smoke-are exposed to some of these toxins. As a matter of fact, there is evidence showing that children residing in households with smokers have higher levels of NNK/nicotine ratios because of THS exposure, when compared to adults [36]. This is very likely because toddlers have the tendency to put objects in their mouth and spend a lot of time “on the floor”. Additionally, toddlers breathe very fast and have thinner skin layers, thereby increasing absorption through the skin [37].

Based on the nature and gravity of THS exposure, coupled with the deficiency in the knowledge regarding its effects, we recently investigated its impact on platelet function and thrombus formation, including under maternal/*in utero* exposure settings and in the context of sex, by employing a validated mouse model. Regarding its *in utero* effects, our results documented that it modulates platelet function and increases the risk of thrombosis in the offspring mice, in a sex-dependent manner [23]. In addition, we have also documented that exposure of “adult” mice to both intermediate and long-term THS (3- and 6-month, respectively) affected the hemostasis function of platelets and rendered mice susceptible to thrombosis [22, 24]. Indeed, there is interest in investigating the effects of THS/cigarette smoking on platelets under short- and long-term conditions [38]. To this end, interestingly, a separate study by our team [25] revealed that even when the exposure is relatively short, namely 1 month, THS still triggers a state of hyperactive platelets and thrombogenesis. Given that the aforementioned studies involved a high dose of THS, specifically 40 cigarettes, we do not know if the same results would be obtained when a low dose, specifically 20 cigarettes, is used (that is, dose dependent effects of THS in the context of platelets). This issue was examined under the 1-month exposure time frame. It is important to note that others have also studied this concept through diverse approaches. For example, a study showed that THS can affect mice *in vivo* by increasing blood sugar levels and delaying wound healing in mice [17, 39].

Before evaluating the platelet functional consequences of low-dose THS, it was essential to confirm that our exposure protocol produced measurable systemic uptake of tobacco-derived toxicants. Therefore, we quantified circulating (serum) cotinine and found significantly elevated levels in THS-exposed mice compared to clean-air controls, verifying that even a reduced THS burden (equivalent to 20 cigarettes) delivers nicotine metabolites into the bloodstream. Moreover, no statistically significant sex differences were detected when comparing the levels in males and females. Importantly, this validation strengthens the interpretability of our subsequent platelet and thrombosis findings, as it demonstrates that the observed phenotypes are linked to quantifiable internal exposures rather than merely environmental contact with THS residues.

Consequently, first, we investigated the effect of 1-month exposure to low dose of THS by carrying out the tail bleeding assay, on male and female mice. Notably, our results showed that the tail bleeding time was significantly shortened in both THS-exposed male and female mice, relative to the clean air-exposed controls. This result is consistent with our recently published data in which we observed that a high dose (40 cigarettes) of THS under 1-month exposures can modulate platelet activity *in vivo*. Based on the aforementioned finding, we hypothesized that these exposed mice may be more prone to thrombosis, which we tested utilizing the widely used ferric chloride-induced thrombosis model. Consistent with our tail bleeding data, our results showed a shortened occlusion time in the THS exposed mice, in both male and female mice. We also investigated the total blood count in the exposed mice, which is a known/standardized test for evaluating general health [40]. We observed no significant differences in both sexes indicating that the platelet and other blood cell life cycle may not have been impacted as a result of the low dose THS exposure. Therefore, the observed phenotype does not appear to involve any changes in blood count, at least under the present experimental conditions.

Based on our *in vivo* findings, we hypothesized that THS produces a hyperactive platelet phenotype that underlies their tendency towards thrombosis. To address this hypothesis, and investigate the mechanism of the observed phenotype, we initially carried out platelet aggregometry experiments. It is important to note that platelet aggregation is shown to be enhanced in smokers, compared to non-smokers [41]. Indeed, the aggregation response was found to be more enhanced in THS exposed platelets regardless of the sex of the mice they were obtained from, and this was the case in response to both ADP and thrombin. These results are all consistent with our previous findings [23, 24], including those done using 40 cigarettes under 1 month of exposure to THS [25]. These data are consistent with work by Hung et al., which investigated the effect of cigarette smoking on platelet reactivity and documented that it enhances platelet aggregation and makes platelets prone to thrombosis [42]. We also investigated the effect of our exposures on both alpha (α) and dense granule secretion. The α-granules’ P-selectin is mostly released upon platelet activation and can be measured with flow cytometric analysis. Interestingly, p-selectin was previously found to be expressed at much higher levels in smokers, when compared to non-smokers [43]. Concerning our low dose THS exposure, we found p-selectin expression levels to be enhanced in response to agonist stimulation in male mice. Furthermore, we also observed a more enhanced dense granule release in both male and female mice, in response to agonist stimulation. These results are consistent with our previous findings [23, 25]. Since we didn’t observe any sex differences in our *in vivo* assays as well as platelet aggregation and dense granule secretion, we decided to carry out the rest of the experiments on only male mice. Next, we investigated the effect of our exposure on agonist-induced integrin activation, which was more enhanced in THS-exposed mice (males), which is in line with our aggregation results.

Platelets stimulated by agonists rapidly expose phosphatidylserine (PS) on their surfaces, which serves as a platform for the assembly of the coagulation cascade [44]. Hence, it serves as a marker of platelet activation *in vitro* [45] and can be measured with flow cytometry. Our results showed higher agonist-triggered PS exposure in the THS exposed platelets. Moreover, our biochemical results revealed enhanced phosphorylation of Akt and ERK in response to low dose THS exposure. It is to be noted that Akt and ERK phosphorylation were also found to be enhanced in other forms of tobacco and/or types of exposures [46, 47].

Finally, we also examined the role of one of the key THS toxicants, NNK as a potential suspect in modulating platelet reactivity. NNK has indeed been identified as one of the major toxic constituents of THS residues, where it accumulates at high levels on indoor surfaces and persists long after active smoking has ceased [17]. Both NNK and NNA (tobacco-specific nitrosamines) have been previously documented to possess mutagenic properties *in vitro*, resulting in DNA damage through transcription and replication impairment, thereby contributing to significant cellular changes, similar to real life THS exposures [48]. Our results suggest that NNK could have a role, at least in part, in promoting thrombosis.

In regard to the possible mechanism of THS induced phenotype, a study carried out on humans have shed light on the potential molecular pathways associated with THS exposure [49]. To this end, THS was found to activate pathways associated with enhanced leukocyte migration and immune cells proliferation-both as a result of upregulation of the Rho family of GTPases-which led to an increase in oxidative stress. Importantly, the RhoA GTPases regulate the reorganization of the actin cytoskeleton, which is a key step in platelet spreading and activation [50], further validating our current and previous findings [25], which showed that THS exposure potentiates platelet spreading.

In a recent study, we also identified a plausible mechanistic pathway by which *in utero* THS exposure promoted a prothrombotic phenotype, by modulating the platelet transcriptome [51]. The results revealed a significant amount of differentially expressed genes and miRNAs controlling platelet signaling and activation as well as other cellular pathways. Bearing in mind that these mice were maternally exposed to THS, it would be worth investigating in the future if direct THS exposure has similar effects on the platelet transcriptome.

Regarding the integrated mechanism underlying the observed phenotype, it is possible that nicotine and tobacco-specific nitrosamines converge on redox and Ca^2+^ pathways that prime platelets for hyperreactivity. In vascular cells, nicotine activates nicotinic acetylcholine receptors (notably α7/α3), raising intracellular Ca^2+^ and driving NOX-dependent oxidative stress and mediating vesicles release [52]. Because human platelets themselves express α7-nAChRs [53], that could provide a direct route to modulate platelet signaling. Consistent with this notion, the α7-nAChR antagonist MG624 was previously found to reverse pathobiologic effects of tobacco toxicants [54], and our unpublished data shows it has the capacity to reverse the prothrombotic phenotype induced by THS. Downstream, platelet NOX isoforms couple ROS to the classical activation machinery, thus, ROS generated by these enzymes promotes platelet activation via the Syk/phospholipase Cγ2/calcium signaling pathway [55]. In parallel, the tobacco carcinogen NNK, which is also an nAChR ligand, is well documented to trigger Ca^2+^ influx through voltage-dependent Ca^2+^ channels [56], a mechanism entirely consistent with modulation of platelet Ca^2+^ handling. Collectively, the aforementioned evidence reinforces the link between tobacco exposure (through its toxic profile), oxidative stress, and heightened platelet responsiveness.

Our study, however, has some limitations, for example, not all experiments could be carried out on both male and female mice due to limited samples, albeit no sex differences were observed in any of the experiments in which male and female mice were used. Similarly, the sample size for our human subjects was very small, and although we took account of subjects’ present medications and health status, we did not take into account the presence of other confounding factors that may impact our results. Finally, our exposure scheme also considered only 1 month (4 weeks) exposure and therefore, potential longer-term effects remain undefined.

Collectively, we document for the first time that 1-month low dose exposure to THS can cause a hyperactive platelet phenotype, as evident by the host of platelet functional studies carried out. Although we did not observe any sex difference with our low dose exposure, it may be possible that a “longer” exposure timeline may show such difference in the observed phenotype, which will be the focus of future research. Nevertheless, our results should serve as the foundation for regulations to restrict tobacco exposure in all its forms.

In summary, our results show that THS exposure negatively impacts health, at least in part, through increasing the risk of occlusive disorders, and involves the toxicant NNK. Also, considering our short exposure time and low dose of cigarettes used, our findings underscore the notion that the negative effects of THS on health do not necessarily require “chronic” exposure, and suggest that it may even be detrimental to people who are subjected to “lower” levels of exposure.

## Data Availability

The original contributions presented in the study are included in the article/supplementary material, further inquiries can be directed to the corresponding author.
